# Improved intraoperative identification of close margins in oral squamous cell carcinoma resections using a dual aperture fluorescence ratio approach: first in-human results

**DOI:** 10.1117/1.JBO.29.1.016003

**Published:** 2024-01-17

**Authors:** Cody C. Rounds, Jaron G. de Wit, Jasper Vonk, Jennifer Vorjohan, Sophia Nelson, Allyson Trang, Brooke Villinski, Kimberley S. Samkoe, Jovan G. Brankov, Floris J. Voskuil, Max J. H. Witjes, Kenneth M. Tichauer

**Affiliations:** a Illinois Institute of Technology, Department of Biomedical Engineering, Chicago, Illinois, United States; b University Medical Center Groningen, Department of Oral and Maxillofacial Surgery, Groningen, The Netherlands; c University Medical Center Groningen, Medical Imaging Center, Department of Nuclear Medicine and Molecular Imaging, Groningen, The Netherlands; d Dartmouth College, Thayer School of Engineering, Hanover, New Hampshire, United States; e Illinois Institute of Technology, Department of Electrical and Computer Engineering, Chicago Illinois, United States

**Keywords:** oral squamous cell carcinomas, oral squamous cell carcinoma, FGS, fluorescence-guided surgery, surgical margins, close margin detection

## Abstract

**Significance:**

Surgical excision is the main treatment for solid tumors in oral squamous cell carcinomas, where wide local excision (achieving a healthy tissue margin of >5  mm around the excised tumor) is the goal as it results in reduced local recurrence rates and improved overall survival.

**Aim:**

No clinical methods are available to assess the complete surgical margin intraoperatively while the patient is still on the operating table; and while recent intraoperative back-bench fluorescence-guided surgery approaches have shown promise for detecting “positive” inadequate margins (<1  mm), they have had limited success in the detection of “close” inadequate margins (1 to 5 mm). Here, a dual aperture fluorescence ratio (dAFR) approach was evaluated as a means of improving detection of close margins.

**Approach:**

The approach was evaluated on surgical specimens from patients who were administered a tumor-specific fluorescent imaging agent (cetuximab-800CW) prior to surgery. The dAFR approach was compared directly against standard wide-field fluorescence imaging and pathology measurements of margin thickness in specimens from three patients and a total of 12 margin locations (1 positive, 5 close, and 6 clear margins).

**Results:**

The area under the receiver operating characteristic curve, representing the ability to detect close compared to clear margins (>5  mm) was found to be 1.0 and 0.57 for dAFR and sAF, respectively. Improvements in dAFR were found to be statistically significant (p<0.02).

**Conclusions:**

These results provide evidence that the dAFR approach potentially improves detection of close surgical margins.

## 1 Introduction

Over 370,000 new cases of oral squamous cell carcinomas (OSCC) were reported in 2020.^1^ The primary treatment for these tumors is surgical resection. ^2^ Yet, owing to the complex anatomy of the head and neck, complete resection of primary tumors is challenging and inadequate margins are observed in up to 40% of cases.^3^^–^^5^ These include both positive margins (tumor within 1 mm of the edge of the resected specimen) and close margins (tumor between 1 and 5 mm of the edge of the resected specimen).^6^^,^^7^ In cases where inadequate margins are found (any <5  mm thick), patients often require intensive adjuvant therapy^8^ because a secondary surgery is typically not possible due to altered anatomy or the proximity of vital structures. In either scenario, patient outcomes are generally worse than if clear margins were obtained on the initial surgery.^9^

Currently, the surgical margin status is determined by histopathology, which takes 3 to 7 days following surgery. This prevents the ability to directly act on results while the patient is still on the operating table. Frozen-section histopathological analysis of at-risk areas can be performed intraoperatively, as results are typically obtained in around 30 min.^10^^,^^11^ However, the staining quality of frozen sections is variable and, more importantly, it requires a subjective decision on part of the surgical team to decide the locations to investigate, leading to sampling error that can result in discrepancies between final pathology in up to 13% of cases.^12^ Such limitations have spurred efforts to develop faster, more accurate methods of assessing the entire margin of the resected specimen intraoperatively.

Over the last decade, significant efforts have been made to establish intraoperative margin assessment using fluorescence molecular guided surgery approaches.^13^^,^^14^ In OSCC, these efforts have predominantly employed imaging agents developed from epidermal growth factor receptor (EGFR)-targeted antibodies. Recently, a phase II clinical trial in 65 OSCC patients showed that EGFR-targeted *ex vivo* fluorescence imaging—referred to in this work as single-aperture fluorescence (sAF)—of entire surgical margins could identify positive margins with a sensitivity of 100%, yet close margin detection yielded a sensitivity of only 70%.^15^ All evidence suggested that the lower detection rates of close margins were caused by the effects of heterogeneous tissue optical properties in the surgical margins.

Here, we introduce a new approach that involves dividing wide numerical-aperture (NA) fluorescence images with narrow NA fluorescence images taken from the same point-of-view, pixel-by-pixel. The resulting ratio image can be shown to provide an estimate of fluorescence depth that is insensitive to tissue optical properties as long as the depth of the fluorescence is considered to be in the sub-diffuse regime.^16^^,^^17^ While previous work employed photon energy bands in the ultraviolet range, limiting the depth of its penetration to <1  mm, here near-infrared (NIR) light is utilized, which contains a much deeper depth of penetration (where the reduced mean free path length is much larger, between 5 mm and 1 cm).^18^

This study compares dual aperture fluorescence ratio (dAFR) against conventional sAF and histopathological assessment of margin thickness in a clinical pilot study, evaluating a total of 12 margins from three patients. We show that dAFR provides accurate information on the surgical margin status in minutes and has the potential to improve the detection of close margins.

## 2 Materials and Methods

### 2.1 Dual Aperture Fluorescence Ratio Imaging System

**Fig. 1:**
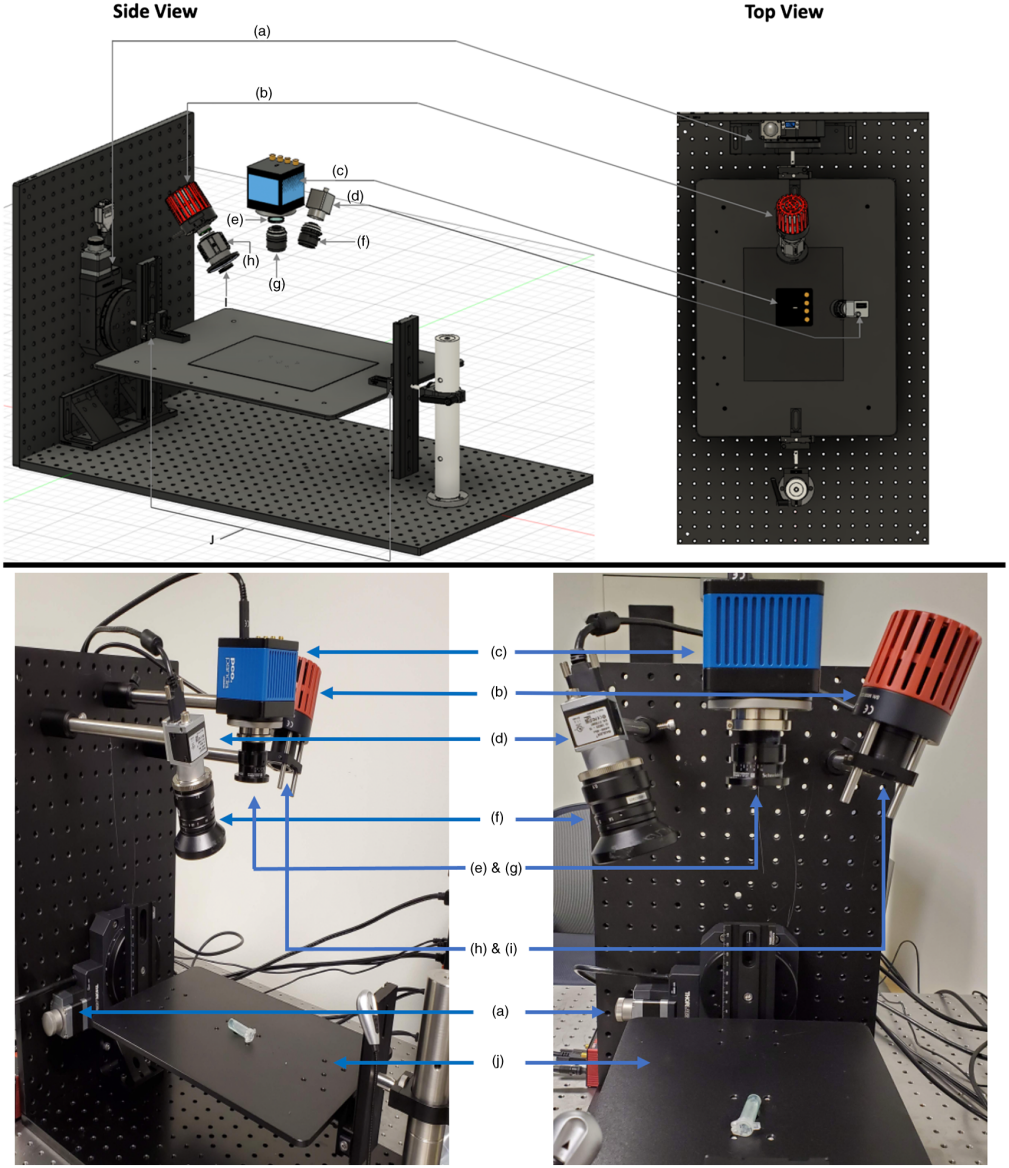
Detailed schematic of the custom widefield imaging system capable of a wide range of numerical apertures at the detector, side view (TOP-left) and top view (TOP-right), and a picture of the true in-house system (BOTTOM)—components: (a) heavy-duty rotation motor, (b) mounted 780 nm LED, (c) 4.2 MP sCMOS fluorescence camera, (d) color camera, (e) 800 nm long pass emission filter, (f) widefield zoom lens, (g) f/2.0-f/16.0 aperture 24 mm widefield lens, (h) LED collimation adaptor, (i) 780±10  nm bandpass excitation filter, (j) height adjustable rail and stage. Note that the clinical adaptation of the system featured the LED in the position detailed in the top schematic.

A dAFR system, capable of capturing fluorescence at a range of numerical apertures, was constructed in house (Fig. 1). The system was fitted with an 800-mW, 780-nm wavelength light-emitting diode (LED) for illumination (Thorlabs, Newton, New Jersey), from which light was passed through a collimation adaptor (Thorlabs, Newton, New Jersey) and a 10-nm bandpass excitation filter centered at 780 nm (Chroma, Bellows Falls, Vermont) to achieve a widefield illumination on the surface of surgical specimens. The power density of the excitation light at the surface of the specimens was $5\;\mathrm{mW}/{\mathrm{cm}}^{2}$, on average, over a field-of-view of 10.5  cm×10.5  cm. Emitted fluorescence light was collected in epi-illumination mode with a 24-mm-focal-length high-resolution visible-near infrared (VIS-NIR) lens (Edmund Optics, Barrington, New Jersey), installed 20 cm from the plane of imaging. The lens was connected to a 4.2-megapixel sCMOS camera (PCO Panda, Kelheim, Germany) and an 800-nm long pass fluorescence filter (Chroma, Bellows Falls, Vermont) was fit between the opening of the lens and the camera sensor. The spatial resolution of the system was set to 205  μm/pixel, isotropic, at a final binned-pixel image size of 512×512, for a field of view of 10.5×10.5  cm.

### 2.2 Clinical Study

#### 2.2.1 Fluorescent antibody

Clinical grade cetuximab-800CW was produced under good manufacturing practice in a dedicated facility at the University Medical Center Groningen (UMCG). A detailed protocol for its synthesis and purification has been described previously.^19^ Briefly, commercially available cetuximab (Erbitux^®^, Eli Lilly and Company, Indianapolis, Indiana) was conjugated with the NIR fluorescent dye IRDye 800CW (LI-COR Biosciences, Lincoln, Nebraska), and the resulting conjugate was purified using PD-10 buffer exchange columns (GE Healthcare, Chicago, Illinois). The purified solution was diluted to 1  mg/mL in sodium-phosphate buffer solution and sterile loaded into vials for later use.

#### 2.2.2 Patients

A subset of six patients with biopsy-confirmed OSCC included in a clinical trial for margin assessment using fluorescence molecular imaging (NCT03134846) were enrolled in the study. Approval for the clinical trial was obtained at the Institutional Review Board of the UMCG (METc 2016/395). The study was performed following the Dutch Act on Medical Research involving Medical Subjects and the Helsinki Declaration (adapted version 2013, Fortaleza, Brazil). Written informed consent was obtained from all patients prior to any study-related procedure.

Two days before primary tumor resection, patients (n=6) were administered an *i.v.* bolus injection of 75 mg of unlabeled cetuximab to minimize off-target receptor binding of labeled cetuximab-800CW,^20^^–^^22^ followed by 15 mg of cetuximab-800CW conjugate 1 h later. Patient vital signs were monitored prior to and after administration of the study drugs for 1 h and if no complications occurred, the patients were discharged. Surgical resection was performed according to the standard of care. Previous work of the UMCG group showed that fluorescence imaging of surgical specimens does not disturb the standard of care.^23^^–^^25^ Short exposure times in the narrow aperture images for the specimens examined from the first three patients resulted in a low signal-to-noise ratio (<5, where the corresponding exposure times were 2, 5, and 10 s), and consequently those samples were removed from analyses, leaving a total of three surgical specimens, which were each analyzed at four distinct locations.

#### 2.2.3 Patient exclusion criteria

Patients were excluded from the study if they presented with a life expectancy of <12 weeks, had a Karnofsky performance status <70%%, had a history of infusion reactions to monoclonal antibody therapies, had a QT prolongation on screening electrocardiogram, were pregnant, had abnormal electrolyte status, were using class IA or III antiarrhythmic drugs, were administered any investigational drugs within 30 days prior to the infusion of cetuximab-800CW, had any episodes within 6 months prior to enrollment (such as myocardial infarction and/or cerebrovascular accident), or presented with any uncontrolled medical conditions (including uncontrolled hypertension, significant cardiopulmonary issues, and/or liver disease).

#### 2.2.4 Specimen basal margin imaging

Immediately following resection, basal surgical margins were imaged using the dAFR system. The height of the stage was adjusted manually to ensure that the basal margin was at the focal plane of the dAFR system (20 cm). Fluorescence signal was first collected at a wide aperture (NA=0.3) followed by a narrow aperture (NA=0.03) at full illumination power. The last three specimens were exposed for 5- and 45-s durations at the wide and narrow apertures, respectively. To maintain specimen moisture, 0.5 ml of 0.9% NaCl solution was dropped onto the specimen at 2-min intervals throughout handling and the excess liquid was carefully blotted off so as to not introduce imaging and motion artifacts in later analysis.

#### 2.2.5 Pathology

**Fig. 2:**
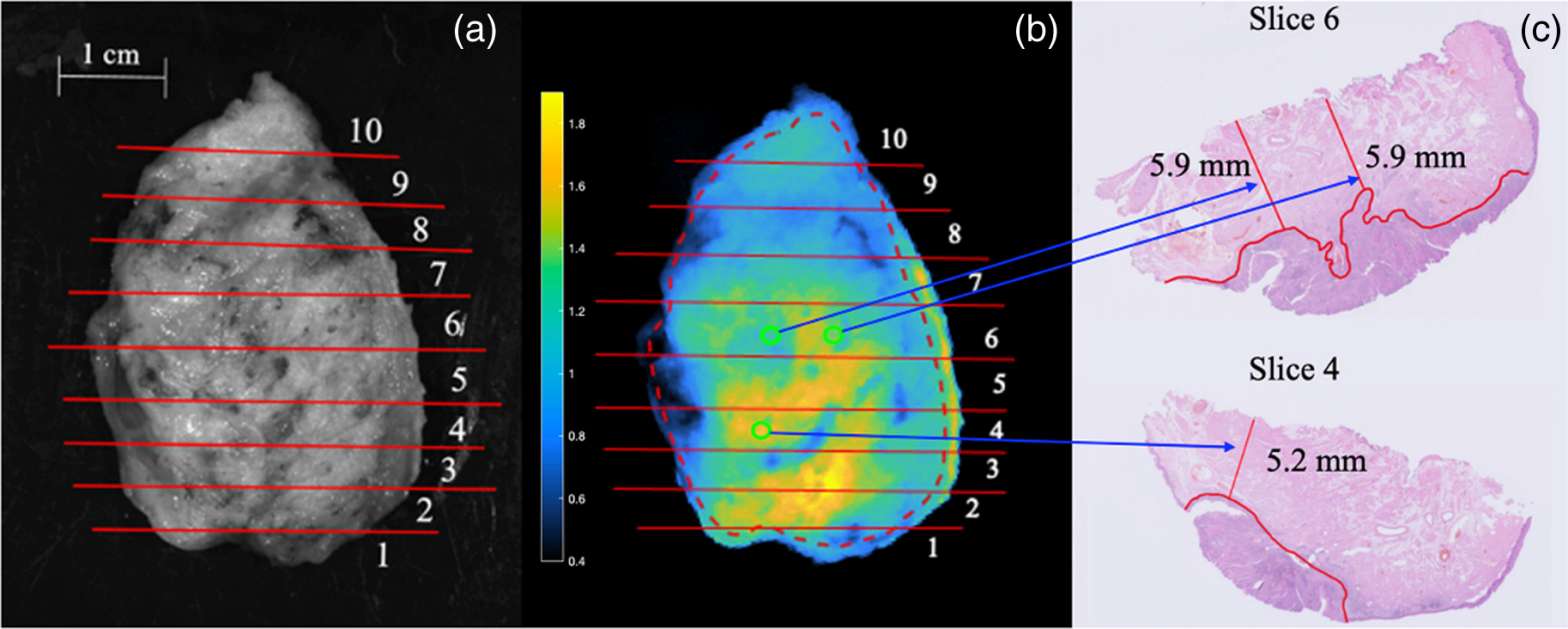
A depiction of the co-registration process employed to evaluate true margin thickness in locations of interest in a whole excised specimen imaged on the deep margin side. (a) The widefield fluorescent image is aligned with the white light image that contains the bread-loaf slice number. (b) Bread-loaf slice number is noted on the widefield image and locations of interest are chosen. Here the dashed red line corresponds to the boundaries of the basal margin. (c) The histology slices of interest are pulled from records and the margin value each location is assessed. Given the bread-loaf slices were 3 to 4 mm thick and the fluorescence signal is being compared with a 4-μm section, and further given the uncertainty in the position of the slice within each noted bread loaf, here each ROI has been drawn an ∼1.5  mm in diameter, with pixel values chosen within the ∼2  mm of uncertainty in true margin location.

Following imaging, the surgical specimens were submitted to the Department of Pathology for routine histopathological assessment. Specimens were formalin-fixed for at least 24 h (depending on specimen size). The margins of the formalin-fixed surgical specimens were inked for orientation purposes. The specimens were then serially sliced into ∼3 to 4 mm thick “bread-loaf” slices. To register the sAF and dAFR images with the corresponding pathology data, white light images of the specimens were taken. The locations and orientations of each bread-loaf slice were noted on the white light image and the image was then aligned with the sAF and dAFR images.^26^ A synopsis of this process is presented in Fig. 2. Each bread-loaf slice was then embedded in paraffin and a 4-μm tissue section was cut from the edge and stained with hematoxylin and eosin (H&E). A pathologist blinded to the fluorescence imaging results inspected the sections to verify the true margin thickness, as per standard-of-care protocol. These measurements were correlated with the sAF and dAFR image values at each corresponding location. For three of the specimens, margin thickness was measured in four locations at the discretion of the pathologist, including the location of the closest margin, yielding a total of 12 margin thickness values that were compared to co-registered regions from the sAF and dAFR images. Owing to the complex and heterogeneous anatomy of the imaged specimens, each margin measurement was considered an independent sample with regard to the statistical analyses.

### 2.3 Image Analyses

#### 2.3.1 Single aperture fluorescence analysis

Single “wide” aperture (NA=0.3) fluorescence images first underwent a flat-field correction by a pixel-by-pixel division of a background image.^27^ This was followed by segmentation of the basal margin from the whole image by the surgical team, where other tissue types (e.g., bone, mucosa) were masked out of the fluorescence images to ensure the image analyses included only the basal margins. Within each basal margin, a background region of interest (ROI) was drawn that included adjacent tissue from the same origin (e.g., connective tissue or muscle). sAF measurements were carried out by dividing the mean pixel value at the locations interest (those delineated by the pathologist) by the background intensity, yielding a signal-to-background ratio (SBR), as described in detail by Ref. 15.

#### 2.3.2 Dual aperture fluorescence ratio analysis

In addition to the wide aperture images used in the sAF analyses, a second image was taken with a “narrow” aperture (NA=0.03), which also underwent a flat-field correction. A 3×3-pixel median filter was then applied to narrow aperture images during post-processing to reduce the contribution of noise in the final analyses. A third “dAFR” image was then created by taking a pixel-by-pixel ratio of the wide aperture divided by the narrow aperture. Measurements from this image were taken directly from the test ROIs with no background normalization.

#### 2.3.3 Statistics

Statistical analyses of the results were done by constructing receiver operating characteristic curves (ROCs) for sAF and dAFR measures of margin status versus true margin depth provided by pathological assessment and areas under the curve (AUCs) were calculated. Statistical comparison between sAF and dAFR AUCs was carried out using the pre-validated iMRMC software,^28^ with which a Student’s t-test was conducted, yielding confidence intervals and p-values to evaluate the existence of statistically significant differences between AUC of the ROC of different datasets. Pearson’s correlation coefficients were calculated to evaluate the correlation between sAF or dAFR values and the true margin thicknesses as determined by histopathology.

## 3 Results

**Fig. 3:**
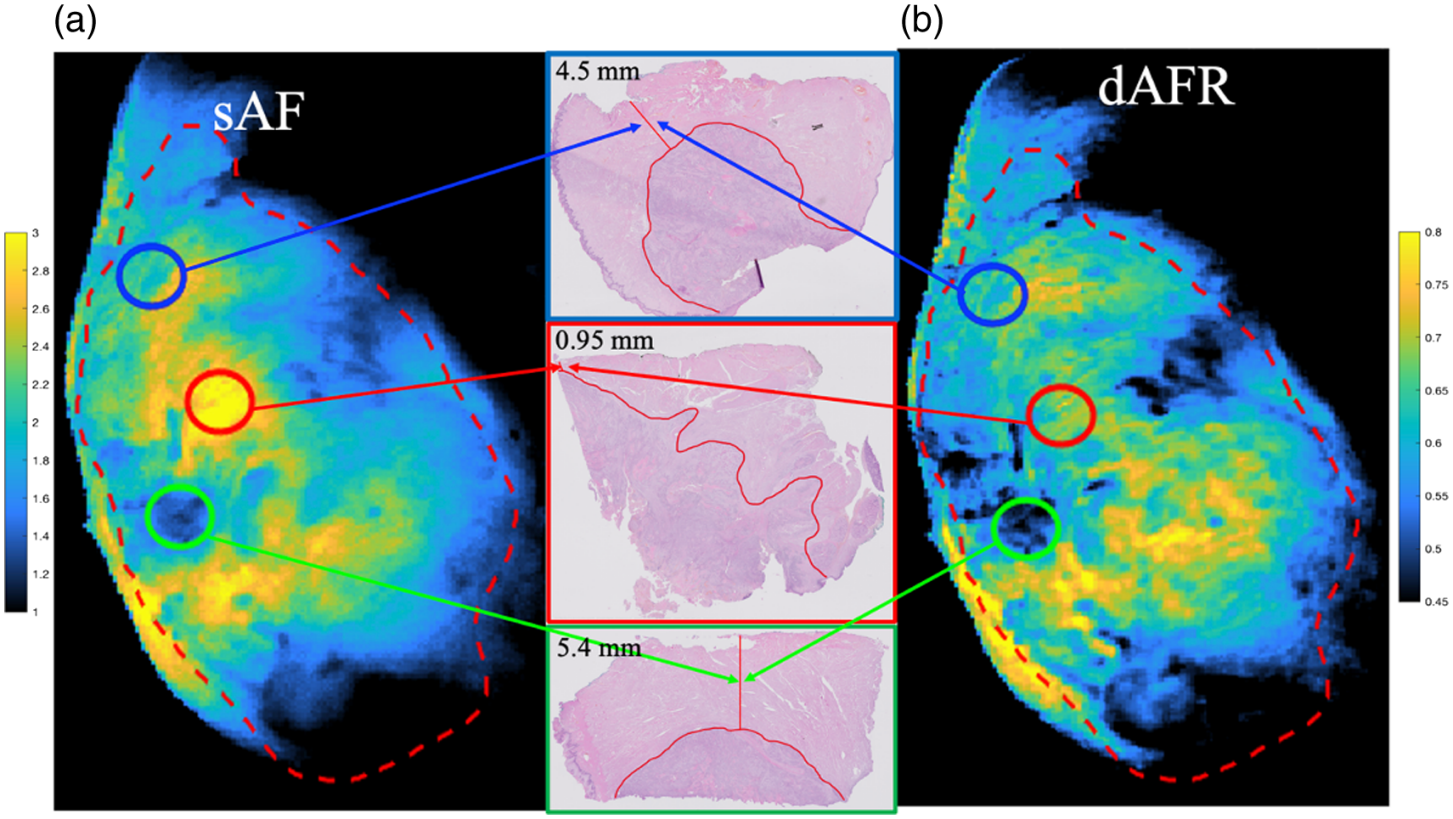
In a whole excised specimen, widefield images of (a) sAF, (b) dAFR, and (c) the associated pathology for each of the noted locations. Here, we note positive margins in red, close margins in blue, and clear margins in green. The boundary of the basal margin is noted with a dashed red line.

**Fig. 4:**
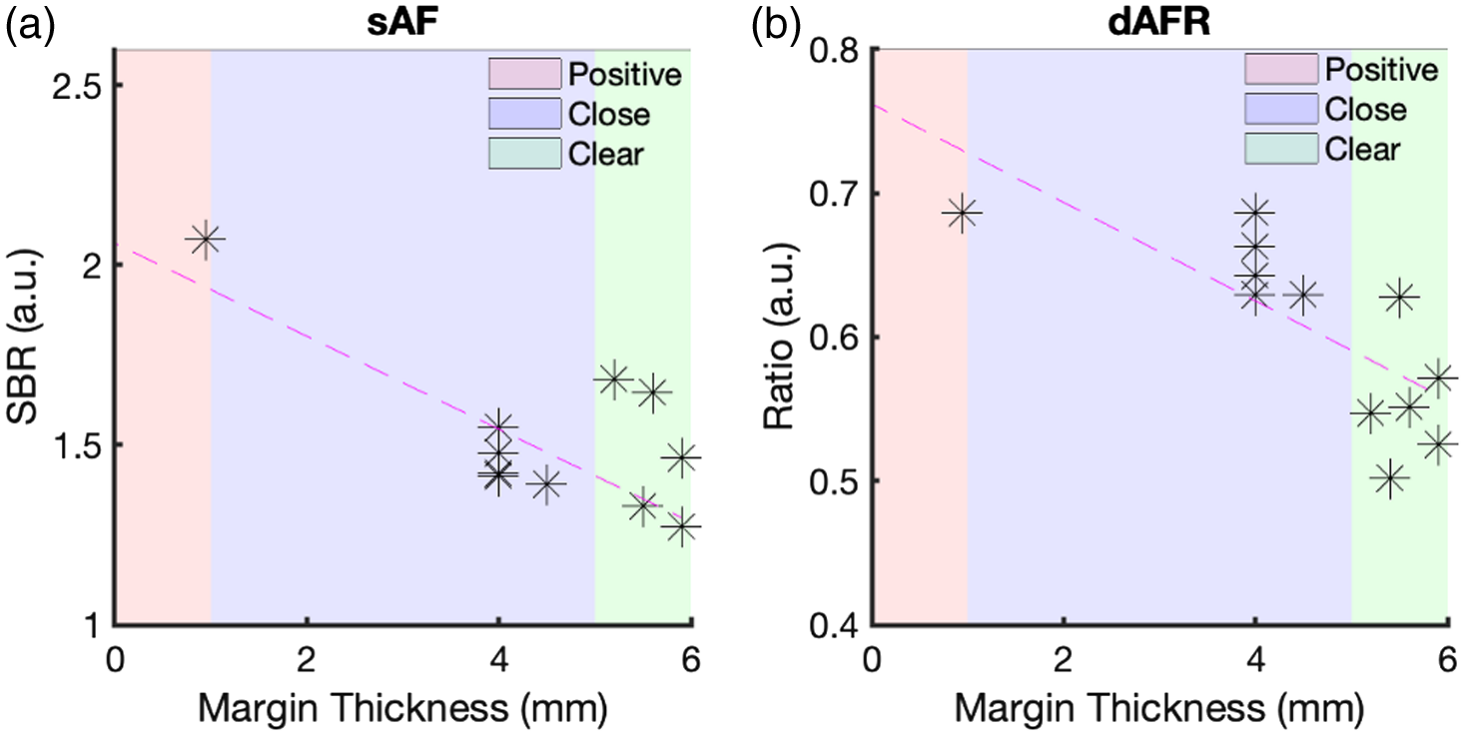
SBR versus true depth of margin for (a) sAF and (b) dAFR.

Patient demographics, clinical data, and histopathological data are provided in the Supplemental Material for the three patient specimens and 12 margins assessed (four margins per specimen). Representative sAF and dAFR images of a specimen alongside its corresponding pathology data are shown in Fig. 3. Figure 4 illustrates the depth versus intensity for both sAF and dAFR measurements. Statistically significant linear correlations were observed between dAFR values and actual margin thickness [Fig. 4(b); r=0.72, p=0.0052], as well as sAF values and actual margin thickness [Fig. 4(a); r=0.61, p=0.022], and the difference in the strength of the correlations was not found to be statistically significant.

**Fig. 5:**
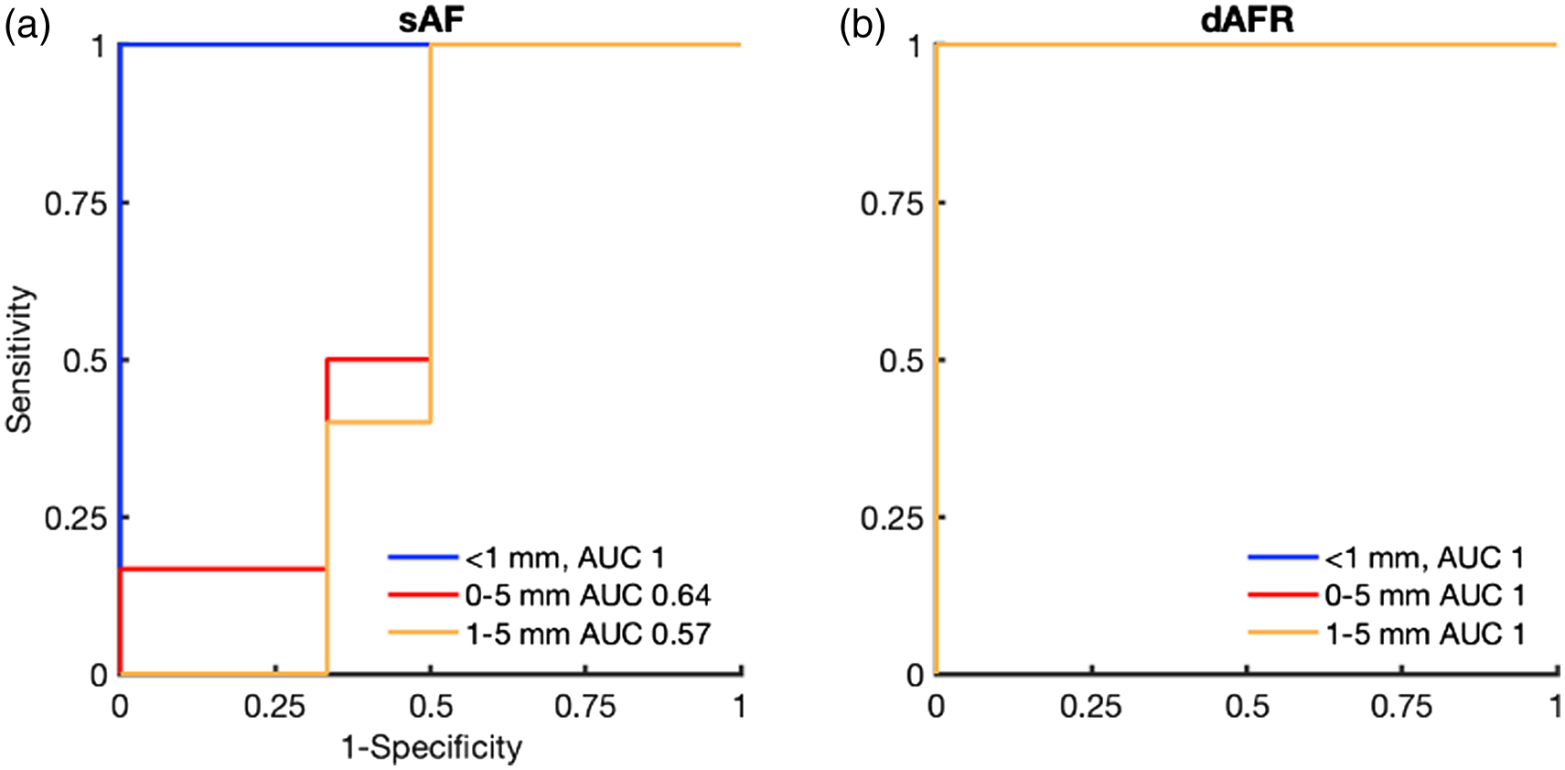
Corresponding ROC analysis for (a) sAF and (b) dAFR. sAF measurements yield AUCs of 1.0 for positive margins (<1  mm), 0.64 for inadequate margins (0 to 5 mm), and 0.57 for close margins (1 to 5 mm). dAFR measurements yield AUCs of 1.0 for all margin classifications.

The dAFR ROC [Fig. 5(b)] curve yielded an AUC of 1.0 for each “positive,” “close,” and “inadequate” (any <5  mm thick) margins. For sAF [Fig. 5(a)], the AUCs of the ROCs were 1.0 for “positive” margins, 0.64 for “inadequate,” and 0.57 for “close” margins. The iMRMC software indicated that the improved detection of “close” margins with dAFR compared to sAF was statistically significant (p<0.02), yielding an estimated 95% confidence interval of the AUC of ROC difference to be between 0.10 and 0.63 if extended to a larger population.

An optimal threshold value was found using Youden’s index for both sAF and dAFR measures.^29^ An SBR of 1.3 was calculated for sAF, while a dAFR value of 0.63 were found to be ideal for discrimination between “close” and “clear” margins. These thresholds yielded a sensitivity of 100% and 100%, a specificity of 50% and 100%, a positive-predictive value of 63% and 100%, and a negative-predictive value of 100% and 100%, for sAF and dAFR metrics, respectively.

## 4 Discussion

This study shows that a dAFR approach provides significant improvements in the detection of close margins compared to standard sAF imaging in OSCC patients injected with cetuximab-800CW prior to surgery (p<0.02) in <1  min of imaging time. This is the first time dAFR has been used in a clinical trial to directly analyze surgical margins, based on these statistically significant improvements in the detection of close margins, could have major implications for intra-operative decision making.

Another key advantage of the dAFR technique over sAF is that no subjective selection of a “background” tissue is required, as is need for the sAF measurements. Rather than normalizing a suspicious signal to a background, dAFR includes a normalization of the wide aperture image with the narrow aperture image, providing an intrinsic point-by-point correction for patient-to-patient or location-to-location differences in exposure time, tracer uptake, and/or heterogeneities in specimen illumination and optical properties. Conversely, sAF analyses of SBR (which help to minimize variability among specimens) require clinical experts to select ROIs in a suspicious region and an appropriate “background” region (one suspected to have a clear margin). This selection process requires a trained and experienced operator, ultimately limiting standardization and reproducibility of results, which is key to ensuring clinical translation of the approach.^30^^,^^31^

While an AUC of the ROC of 1.0 for close margin detection with dAFR is impressive, this is a small sample size and so expansion of this work is required for a more definitive estimation of detection accuracy.^32^ Yet, although the study was limited to three patient specimens, a more representative sample size of 12 was approximated by evaluating each specimen at four distinct locations. While these are technically “within-subjects” variables, the optical property heterogeneity within any single sample is expected to be representative of the variability across multiple samples owing to the complex anatomy of the specimens imaged. Furthermore, given the statistical significance of the results presented here from a head-to-head comparison with the state-of-the-art SBR of a single wide aperture image, these results are encouraging and merit expansion and further investigation in future work.

In terms of evaluating positive margin detection, both sAF and dAFR were able to distinguish the single identified positive margin in the data set with an AUC of the ROC of 1.0; however, since there was only a single case, it was not possible to evaluate these results statistically. It should be noted that in the larger sAF study,^15^ the AUC of the ROC for positive margin detection accuracy was found to be 0.91, and it is not expected that dAFR need to outperform sAF for positive margin detection for it to be clinically useful, as long as it provides a better detection of close margins, as observed in this pilot study.

While the potential of dAFR for improving intraoperative margin assessment of close margins is evident, this pilot study has some limitations, including the potential for low photon budget when imaging at the narrow aperture and the ability to adapt dAFR to specimens with highly heterogeneous surface topographies. In the context of a low photon budget, the PCO camera used here, while capable of imaging with a relatively low read-noise of $2.3\;\mathrm{rms}\;e^{-}$, was only capable of generating sufficient contrast in the narrow numerical aperture images with long exposure times (at least 45 s per image). To combat this, one could use a more advanced qCMOS detector, which can be capable of “single photon counting” sensitivity and could yield a greater than four time improvement in SNR and allow for shorter imaging times. Second, a more powerful light source would also improve the system used in this work, which employed a light source two to three orders of magnitude lower than the ANSI safety limit. With improved photon budgets at narrow apertures, it is conceivable that the relationship between dAFR and margin thickness can be improved substantially either through greater SNR or enhanced narrowing of the narrow-aperture image, which is also expected to improve discrimination/correction, as it can reject more highly scattered photons. A full optimization of what is possible in clinically relevant imaging times and without damaging samples is ongoing using Monte Carlo simulations.^33^

To partially account for heterogeneous margin surface topographies, the designed system was fitted with a heavy-duty rotation motor to which a height-adjustable stage was attached. The height-adjustable stage allowed for imaging of specimens that vary from 1 to 10 cm thickness while keeping the surface of the specimen in the focal plane of the lens (where depth of field was ∼1 and ∼3  cm, respectively, for the narrow and wide aperture images). It should also be noted that the heavy-duty rotation mount presents the ability to image the surface of the specimen at multiple angles, opening the possibility to correct for surface topography^34^ or for limited-angle tomography^35^ that could be combined with dAFR to further improve depth estimation in future.

## Data Availability

All code, data, and materials used in this project and analysis can be obtained by direct email to the corresponding author, Dr. Kenneth M. Tichauer at ktichaue@iit.edu.
