# Supplementary material for: Improved intraoperative identification of close margins in oral squamous cell carcinoma resections using a dual aperture fluorescence ratio approach: first in-human results
Source: J Biomed Opt. 2024 Jan 17;29(1):016003. doi: 10.1117/1.JBO.29.1.016003 (PMC10793906; doi:10.1117/1.JBO.29.1.016003)
Supplement: Supplementary file 1 [file JBO_029_016003_SD001.pdf]

Supplemental Information

Patient demographic & clinical data

ICON	Patient	Full protocol	Sex (0=M, 1=F)	Operation Date	Age during OR	Weight (KG)	Height (cm)	BMI (kg/m^2)	Smoking (0=no, 1=yes, 2=history of smoking)	Alcohol (0=no, 1=<2units/day 2=>2units/day 3=history of >2units/day)	ACE27 score
**90**	71	63	M	10/20/21	39	87	177	27.8	History	2	0
**91**	72	64	M	11/24/21	52	78	185	22.8	History	3	3
**92**	73	65	F	11/26/21	77	65	165	23.9	History	1	1

Patient histopathology data

ICON	T-stage	T-stage simplefied	N-stage	M-stage	Location	Lateralisation	Diameter(mm)	Thickness (mm)	Invasiondepth (mm)	differentation	Growth pattern	Lymfangioinvasie	Perineural growth	Boneinvasio	Dysplasia near tumor (	Ammount of biopsies taken
**90**	T2	T2	0	0	Tongue	Left	25	5.3	5.9	Poor	Invasive & pushing	No	No	No	Severe	0
**91**	T4a	T4	2b	0	Tongue	Left	60	27.2	27	Poor	Pushing	Yes	Inside Tumor	No	No	0
**92**	T1b	T1	0	0	Mandibula	Left	6	0.9	1.3	Poor	Pushing	No	No	No	Mediocre	0
